# Association of MIF, but not type I interferon-induced chemokines, with increased disease activity in Asian patients with systemic lupus erythematosus

**DOI:** 10.1038/srep29909

**Published:** 2016-07-25

**Authors:** K. L. Connelly, R. Kandane-Rathnayake, A. Hoi, Mandana Nikpour, E. F. Morand

**Affiliations:** 1 Centre for Inflammatory Diseases, School of Clinical Sciences at Monash Health, Faculty of Medicine, Nursing and Health Sciences, Monash University, Melbourne, Australia; 2 Department of Medicine and Rheumatology, The University of Melbourne at St. Vincent’s Hospital, Melbourne, Australia

## Abstract

Ethnicity is a key factor impacting on disease severity in SLE, but molecular mechanisms of these associations are unknown. Type I IFN and MIF have each been associated with SLE pathogenesis. We investigated whether increased SLE severity in Asian patients is associated with either MIF or Type I IFN. SLE patients (n = 151) had prospective recording of disease variables. Serum MIF, and a validated composite score of three Type I IFN-inducible chemokines (IFNCK:CCL2, CXCL10, CCL19) were measured. Associations of MIF and IFNCK score with disease activity were assessed, with persistent active disease (PAD) used as a marker of high disease activity over a median 2.6 years follow up. In univariable analysis, MIF, IFNCK score and Asian ethnicity were significantly associated with PAD. Asian ethnicity was associated with higher MIF but not IFNCK score. In multivariable logistic regression analysis, MIF (OR3.62 (95% CI 1.14,11.5), p = 0.03) and Asian ethnicity (OR3.00 (95% CI 1.39,6.46), p < 0.01) but not IFNCK were significantly associated with PAD. These results potentially support an effect of MIF, but not Type I IFN, in heightened SLE disease severity in Asian SLE. The associations of MIF and Asian ethnicity with PAD are at least partly independent.

Systemic lupus erythematosus (SLE; lupus) is a chronic autoimmune disease characterised by immunologically-mediated inflammatory activity across multiple organ systems and the potential for irreversible end organ damage^1^. Ethnic differences in disease expression have been widely noted in SLE. For example, Asian ethnicity has been linked to a more severe SLE phenotype, including higher frequency of renal disease, and serological manifestations (including anti-dsDNA, anti-Ro and anti-Sm positivity), and higher disease activity^2^. These findings, largely derived from comparisons of independent cohorts, were replicated in a single setting multi-ethnic cohort comprising predominantly Asians and Caucasian SLE patients at the authors’ centre^3^. In this study, markers of disease severity including frequency of persistently active disease (PAD) were significantly higher among Asian patients. The biological basis of this disparity is unknown, but variations in biological determinants of disease outcome such as pro-inflammatory cytokine expression are potential candidates.

The pathogenesis of SLE is complex, and a combination of genetic and environmental factors are likely to play a role^1^. Abnormalities across the immune system have been reported, including altered T and B cell function, the presence of pathogenic autoantibodies, increased macrophage recruitment and activation, and the over-expression of pro-inflammatory cytokines. For example, type I interferons (IFN) such as IFN-α, and macrophage migration inhibitory factor (MIF), are cytokines that have been demonstrated in murine and human studies to be implicated in the pathogenesis of SLE, and have been the subject of clinical trials in SLE. Activation of the Type I interferon (IFN) system, for example as evidenced by a pattern of increased transcriptional activity of selected IFN-inducible genes, has been detected in approximately half of adult SLE patients^4^,^5^,^6^. Serum concentrations of chemokines induced by Type I IFN, namely CCL2, CXCL10 or CCL19, are also elevated in SLE, and correlate tightly with IFN-inducible gene expression^7^,^8^. Monoclonal antibodies against Type I IFN pathways are currently in clinical trials in SLE^9^,^10^, but which patients are most likely to benefit from such therapies is not established. Similarly, the proinflammatory cytokine macrophage migration inhibitory factor (MIF) mediates a broad spectrum of pro-inflammatory functions in diseases including SLE^11^,^12^. MIF is produced by many immune cells, including macrophages and dendritic cells, and its release may be stimulated by the presence of immune complexes^13^. Animal studies have suggested an important functional role for MIF in models of SLE, with MIF deficiency conferring significant protection in two different lupus mouse models^14^,^15^. In the MRL/*lpr* mouse for example, *Mif* deletion led to almost complete protection from crescentic glomerulonephritis and a 50% improvement in survival^14^. In human SLE, serum MIF concentrations are raised compared to healthy controls^16^, and high expression polymorphisms of the *MIF* gene have also been linked to SLE disease susceptibility and severity^17^.

The association of cytokines such as IFN-α and MIF with SLE phenotype raises the possibility that factors such as these are implicated in the increased SLE severity observed in Asian patients. The aim of this study was to investigate the associations of serum concentrations of MIF and a marker of Type I IFN activity with ethnicity and measures of disease severity, in a prospective, multi-ethnic lupus cohort. Our findings suggest that increased disease activity in Asian SLE patients is associated with increased serum MIF, but not with Type I IFN.

## Results

### Patient characteristics

This study included 151 SLE patients, whose characteristics are outlined in Table 1. The study cohort was 84% female, with a median age at enrolment of 42 years, and 40% of patients were of Asian ethnicity; non-Asian patients were of majority European descent (Table 1).

Of 151 patients, 126 had clinical data available from at least one occasion subsequent to the baseline visit. Over the course of the study period (median follow-up 2.8 years, IQR 1.4, 3.7), 92 patients (62%) had episodes of persistently active disease (PAD), 74 patients (51%) had time-adjusted mean disease activity (AMS) > 4, and 41 (27%) accrued irreversible organ damage (defined as increase in SLICC-DI score ≥ 1 unit/s) (Table 1).

MIF was detectable in all samples, with a range from 20 pg/mL to 111 ng/mL (Table 1); the median serum MIF concentration was 6.7 ng/mL (IQR 4.16, 12.1) (Table 1). The chemokines contributing to the IFNCK score were detectable in all samples (CCL2: range 3.3–7853 pg/mL; CXCL10: range 3.81–11227 pg/mL; CCL19: 5.6–10981 pg/mL). The median IFNCK score was 0.27 (IQR 0.16, 0.57) (Table 1). MIF concentrations and IFNCK scores are shown in Supplementary Figure 1 and were found to be highly correlated (r = 0.78, p < 0.01).

### Clinical characteristics and ethnicity

We next compared clinical characteristics between Asian and non-Asian patients. Asian patients were significantly younger at diagnosis, had significantly higher rates of high titre (>1:1280) ANA, had significantly higher rates of detectable antibodies to extractable nuclear antigens, and were more likely to have positive anti-dsDNA antibody titres (P = 0.05) (Table 2). There were no significant differences between Asian and non-Asian patients in gender, the number of ACR SLE classification criteria, baseline SLICC-SDI, or serum complement C3 or C4 at baseline (Table 2). Higher disease activity in Asian patients was signified by the observation that PAD was significantly more frequent among Asian patients (Table 2). Analysis of the factors associated with PAD detected that Asian ethnicity was a significant predictor, with an odds ratio of PAD more than three times greater in univariable analysis (Table 3). Despite a similar length of follow up, Asian patients had significantly higher frequency of clinic visits, and were more likely to be receiving glucocorticoid therapy, both surrogate indicators of disease activity (Table 2). Baseline SLEDAI–2K, and AMS over the period of observation, were also numerically higher in Asian patients although these differences did not reach statistical significance (Table 2).

### MIF and IFNCK associations with disease activity

Associations between serum MIF and patient characteristics were first evaluated using univariable logistic regression analysis. Associations between serum MIF and disease related variables including anti-dsDNA and ENA autoantibodies, serum complement levels, SLEDAI–2K and SLICC-SDI at baseline were not significant, and baseline MIF was not predictive of damage accrual (∆SLICC-SDI > 0) (data not shown). In contrast, univariable analysis indicated that high baseline serum MIF was statistically significantly associated with PAD, such that the odds ratio of PAD in patients with MIF in the highest quartile was more than four times greater than those patients in whom MIF levels were in the lowest quartile (OR = 4.6, [95% CI: 1.49–14.2], p < 0.01) (Table 3). The association between high serum MIF and PAD remained statistically significant after adjusting for IFNCK scores and ethnicity (Table 4), with an odds ratio of 3.62 (1.14–11.5). In univariable analysis high baseline MIF was also statistically significantly associated with high disease activity as measured by AMS > 4 (OR 2.89; 95% CI1.07–7.77, p = 0.04), but did not remain significant after adjusting for IFNCK scores and ethnicity. With regard to organ-specific disease activity, high levels MIF was associated with cutaneous activity in univariable analysis. Levels of MIF were not significantly associated with glucocorticoid use.

Univariable logistic regression analyses indicated that high IFNCK score was also statistically significantly associated with PAD, with an odds ratio of 2.67 (1.04–6.87, p = 0.04) (Table 3). The association of high baseline IFNCK scores with PAD did not remain significant after adjusting for MIF and ethnicity (Table 5). Similarly, high IFNCK scores were significantly associated with high disease activity (AMS > 4), with an odds ratio for high disease activity in patients with baseline IFNCK score in the highest quartile of 2.56 (95% CI: 1.03, 6.37, p = 0.04); this did not remain significant after adjusting for MIF and ethnicity in multivariable analysis (not shown). IFNCK score was not predictive of damage accrual (∆SLICC-SDI > 0) but was significantly associated with low complement C3 (data not shown). With regard to organ-specific disease activity, high IFN-CK was associated with musculoskeletal activity in univariable analysis. Levels of IFN-CK score were significantly increased in association with glucocorticoid use.

### Cytokine associations with ethnicity

Asian patients had significantly elevated MIF levels. The geometric mean of MIF was 82% higher in Asians (9.87 ng/mL, 95% CI 7.31–13.4) when compared to Caucasians (5.42 ng/mL 95%CI 4.25–6.92) (p < 0.01) (Table 2). In contrast, there was no significant difference in IFNCK score between ethnicities. Given the association of MIF and IFNCK with high disease activity as measured by PAD, we performed further analyses to determine whether these pro-inflammatory biomarkers could explain associations between Asian ethnicity and PAD. The significant association between Asian ethnicity and PAD in univariable analysis (Table 3) remained significant after controlling for serum MIF and IFNCK (Tables 4 and 5). The association between high MIF and PAD was slightly weakened after adjusting for ethnicity and IFNCK score, but remained statistically significant (OR 3.62, [95% CI 1.14, 11.5], p = 0.03, Table 4). In contrast, the association between IFNCK and PAD was non-significant after adjusting for ethnicity and MIF (Table 5). These results suggest overlapping but partially independent associations of Asian ethnicity and serum MIF, but not IFNCK, with the presence of PAD in patients with SLE. There were no significant ethnicity-based variations in the associations of MIF and IFN-CK with musculoskeletal and cutaneous disease activity, or other organ-specific manifestations (data not shown).

## Discussion

Ethnicity is one of many variables repeatedly reported to impact on disease expression in SLE. African American, Hispanic, and Asian ethnicity have each been shown to be associated with higher levels of disease activity and/or susceptibility to SLE^18^. For example, studies comparing cohorts in separate locations have shown that Asian ethnicity is associated with increased renal disease, autoantibody manifestations and overall disease activity^2^, and these finding have been confirmed in a single multi-ethnic cohort^3^. While increasing numbers of polymorphisms in key immune response pathways, including Type I IFN pathways, have been associated with SLE susceptibility^19^, polymorphisms associated with SLE in Asian and European SLE patients overlap^19^,^20^. To date, no study has investigated whether differences in serum cytokine levels are implicated in phenotypic differences in Asian and non-Asian SLE. In this study we investigated whether MIF or Type I IFN, both of which have been implicated in SLE pathogenesis, are pertinent to increased disease activity in Asian patients with SLE. The chief findings of this study, undertaken in a single centre multiethnic cohort, are that increased disease activity in Asian SLE patients, as measured by PAD, is associated with increased serum MIF but not Type I IFN induced chemokines, and that increased PAD in Asian SLE patients is also driven by factors independent of these two pathways.

This study confirms a strong association of Asian ethnicity with indicators of SLE severity, including glucocorticoid use, high titre autoantibodies and disease activity, as measured by PAD. PAD is defined when SLEDAI–2k is greater than four on two or more occasions sequentially, excluding serology alone^21^, and therefore requiring the inclusion of sustained clinical disease activity. These findings are aligned with those of an earlier study from this centre^3^ and with previous studies comparing SLE manifestations in separate Caucasian and Asian cohorts^22^. While some studies have highlighted economic and health system differences in contributing to divergent SLE outcomes in different regions^23^, the current study was undertaken in a single centre based in a public hospital in a universal healthcare setting^24^, consistent with a biological basis for the difference in disease severity in SLE patients of Asian, compared to non-Asian descent.

We selected MIF and the Type I IFN pathway for the current investigation based on the fact that MIF and Type I IFN have each been demonstrably associated with the pathogenesis of SLE in both murine and human studies, and are the subject of clinical trials in SLE. MIF activates intracellular signaling events which lead to the upregulation of genes involved in inflammation^25^,^26^, increases leukocyte recruitment^27^,^28^, T cell activation^29^ and B cell proliferation and survival^30^. Increased MIF has been demonstrated in human SLE^16^, polymorphisms in the *MIF* gene promoter have been associated with SLE susceptibility^17^, and MIF deletion or inhibition abrogates disease expression in murine models of SLE^14^,^15^. Similarly, multiple gene variants found in loci connected to the Type I IFN system have been linked with SLE^19^ and multiple studies indicate the presence of increased Type I IFN activity in SLE^6^,^31^. A broad range of effects of Type I IFN are implicated in SLE pathogenesis^32^, and these effects may explain the reproducible association of Type I IFN activity with a more severe disease phenotype in SLE, including increased disease activity, nephritis and serological abnormalities^31^,^33^,^34^. The Type I IFN-induced chemokines CCL2, CCL19 and CXCL10 have been demonstrated to correlate highly with other measures of the Type I IFN signal and with certain measures of disease activity in SLE^7^,^8^; notwithstanding this, measurement of other IFN-induced targets in future studies, including mRNA expression of IFN-induced genes and IFN-initiated signaling pathways may provide information not captured in the current study.

In the current study, both MIF- and IFN-induced chemokines were detectable in 100% of patients, in contrast to many other cytokines that are only detectable in a subset of cases^35^,^36^,^37^. High serum MIF was associated with markers of disease activity. In univariate analysis, MIF was significantly associated with PAD and AMS > 4, and an independent association of MIF with PAD was confirmed in multivariate analysis, controlling for IFNCK score. Previous studies of serum MIF and disease activity in SLE have been small and cross-sectional in nature, but have suggested associations of serum MIF with disease severity^16^,^38^. The lack of association of MIF with serological determinants of disease activity corresponds with findings in mouse models of SLE wherein profound protective effect of MIF inhibition occurred in the absence of changes in autoantibody titres^14^.

The hypothesis that MIF is implicated in the association of Asian ethnicity with high disease activity was supported. Serum MIF concentrations were significantly higher in Asian compared to non-Asian SLE patients, and univariable analysis revealed that high MIF concentrations were associated with a three-fold risk of PAD. Multivariable analysis revealed that the association of each of MIF and Asian ethnicity with PAD was somewhat attenuated when adjusted for the other, but remained significant for both. This implies that the association of Asian ethnicity with active disease is at least partly independent of MIF, and similarly that the association of MIF with active disease is not restricted to the higher MIF levels in Asian SLE patients. However, these results do suggest for the first time a therapeutically addressable target for reducing heightened disease activity in Asian SLE patients, suggesting that clinical trials of anti-MIF therapies in SLE^39^ could be considered particularly appropriate in this subgroup. While polymorphisms in *MIF* have been associated with SLE in both Asian and non-Asian cohorts, differences in MIF levels or MIF genotype between ethnicities in patients with autoimmune disease have not been previously reported.

This study also demonstrated an association between Type I IFNCK score and high disease activity as measured by PAD in univariate analysis. High IFNCK score was also significantly associated with low levels of complement C3 and there was a trend towards an association with anti-dsDNA and ENA positivity. This is in keeping with previous studies of the IFN signature where high IFN activity was associated with anti-dsDNA autoantibodies and low complement but not measures of clinical disease activity^40^. The association of IFNCK with PAD was not significant after adjusting for covariates, and there was no association between IFNCK score and Asian ethnicity, implying that this pathway is not involved in the higher disease activity observed in Asian SLE patients.

There are certain limitations to the interpretation of the current study. It was undertaken in a single centre cohort, although this allowed more direct comparison of ethnicities in the same care context. The measurement of IFN pathway activation used here, based on Type I IFN-induced chemokine concentrations, has not been validated in Asian SLE patients and it remains possible that an alternative measure of Type I IFN pathway activation, such as transcriptomal analysis, would reveal associations between Type I IFN activation and increased PAD in Asian SLE patients. It is also important to note that studies of longitudinal variation in serum MIF and IFN-CK with longitudinal changes in SLE status could provide additional insights. In addition, measurement of serum MIF and IFN-CK relative to levels in healthy controls might provide a different method with which to stratify levels of these proteins among SLE patients.

In conclusion, we have demonstrated a novel association between MIF, high disease activity, and Asian ethnicity in SLE. Asian ethnicity was associated with increased disease activity and increased serum MIF, but the associations of MIF and Asian ethnicity with disease activity were partly independent. In contrast, Type I IFN inducible chemokines were not increased in Asian SLE patients and an association with high disease activity as measured by PAD was non-significant after adjusting for covariables. This does not exclude an association of Type I IFN pathways with PAD in Asian SLE patients as biomarkers of IFN activity other than chemokine score were not measured. Nonetheless, these findings add to the growing literature supporting a pathogenic role for MIF in SLE, and suggest further investigation of MIF as a therapeutic target in subsets of SLE patients of Asian descent.

## Methods

### Study design and participants

Data were prospectively acquired between June 2007 and January 2012 from patients who attended the SLE Clinic at Monash Medical Centre, a tertiary referral public hospital in Melbourne, Australia^35^,^36^,^37^. Eligible patients fulfilled the American College of Rheumatology (ACR) criteria for the classification of SLE^41^, were over 18 years of age, and provided written informed consent. Birth date, gender, year of disease onset and self-assigned ethnicity^3^ were recorded at baseline. Patients were included in the current study if they had complete clinical data and a matched serum sample available at a given visit. Clinical data from all subsequent visits during the study period were collected. Ethics approval for this study was obtained, and methods carried out, in accordance with the Monash Health Human Research Ethics Committee.

### Patient information

Patients were seen at 3–6 monthly intervals, or more frequently according to clinical need. At each clinic visit, disease activity was documented using the 2000 modification of the SLE disease activity index (SLEDAI–2K)^42^. Persistently active disease (PAD) was assigned as described^21^, defined as ≥2 consecutive visits with SLEDAI–2k ≥ 4, excluding SLEDAI scores comprised only of serological parameters (low complement and raised anti-double stranded DNA antibody titres). A measure of disease activity over time was generated using the adjusted mean SLEDAI (AMS)^43^, and AMS > 4 was considered high disease activity. Organ-specific disease activity was measured by grouping domains of the SLEDAI–2k according to the organ system affected and assigning disease as active in a given system if any relevant SLEDAI–2k domain was scored. Disease-related damage was assessed at baseline and annually using the Systemic Lupus International Collaborating Clinics Damage Index (SLICC-SDI)^44^. Autoantibody positivity was documented at baseline and included a record of ANA titre, anti-double stranded DNA (anti-dsDNA) positivity and the presence of antibodies to a range of extractable nuclear antigens (ENA) including RNP (ribonucleoprotein), Sm, Ro, and La.

### Measurement of serum concentrations of MIF and IFN-induced chemokines

Patient serum samples were obtained and stored at −80 °C until use, as described^35^,^36^,^37^. Activation of Type I IFN pathways was assessed by measurement of three Type I IFN inducible chemokines (CCL2, CXCL10 and CCL19) as described by Bauer^7^. Concentrations of serum MIF and CCL2, CXCL10 and CCL19 were determined in each sample using sandwich ELISA, as previously described^35^,^36^. Briefly, 96-well plates (Immunoplates, Nunc, Roakilde, Denmark) were coated with primary antibody (anti-human MIF, CCL2, CXCL10 or CCL19; R&D Systems, Minneapolis, MN, USA) and incubated overnight before being blocked by 1% bovine serum albumin. After washing, recombinant human protein standards and serum samples were added in duplicate and incubated overnight. Binding was detected using a biotinylated goat anti-human antibody (R&D Systems) and streptavidin conjugated to horseradish peroxidase (Silenus, Melbourne, Australia). Colour was developed with 3,3′5,5′-tetramethylbensidine (Sigma, Sydney, Australia) and read at 450 nm.

In order to integrate the results obtained for the three Type I IFN regulated chemokines, a composite IFNCK score was derived for each sample, in the manner validated by Bauer *et al*.^8^. Concentrations above the 95th centile for each chemokine were assigned a value of one, with the remaining concentrations scaled to this percentile. Scaled values for each chemokine were then added to produce a final IFNCK score ranging from 0 to 3.

### Statistical Analysis

All statistical analyses were performed using Stata version 13.1 (StataCorp, College Station, Texas, USA). Continuous variables were described as median (interquartile range [IQR], range) and compared using Wilcoxon rank sum tests. Categorical variables were described as frequency (%) and compared using Chi-square tests. As values of both were positively skewed, serum MIF concentrations and IFNCK scores were reported as geometric means with corresponding 95% confidence interval (CI), and for linear regression analyses were categorized as quartiles. Univariable linear regression analyses were initially used to evaluate associations between MIF and IFNCK score respectively with patient variables. Variables with p-value < 0.1 in the univariable linear regression analyses were included in multivariable linear regression models. A p-value ≤ 0.05 was considered statistically significant. In addition, univariable and multivariable logistic regression analyses were carried out to evaluate whether baseline serum MIF and IFNCK score were associated with disease outcomes including PAD, AMS and change in SLICC-SDI. Potential confounding variables were included in the multivariable logistic regression models on the basis of their association with MIF/IFNCK score as well as the clinical outcome.

## Additional Information

**How to cite this article**: Connelly, K. L. *et al*. Association of MIF, but not type I interferon-induced chemokines, with increased disease activity in Asian patients with systemic lupus erythematosus. *Sci. Rep*. **6**, 29909; doi: 10.1038/srep29909 (2016).

## Supplementary Material

Supplementary Figure S1

Supplementary Table S1

Supplementary Table S2

## Figures and Tables

**Table 1:** Characteristics of study population.

	Total (n = 151)	
	Median	[IQR] (range)
Age at diagnosis (years)	42	[32, 53] (20, 79)
Disease duration at recruitment (years)	8.6	[4.6, 16.6] (1.5, 36.6)
Length of follow-up (years)	2.8	[1.4, 3.7] (0, 4.6)
Number of visits	5	[2, 10] (1, 27)
ACR criteria	5	[4, 6] (4, 9)
SLEDAI–2K (baseline)	4	[2, 8] (0, 20)
Time adjusted mean SLEDAI (AMS)	4.1	[2.3, 6.3] (0, 15.1)
SLICC-SDI (baseline)	0	[0, 1] (0, 7)
	**n**	(**%**)
Females	127	(84%)
Ethnicity		
Asian	60	(40%)
Caucasian	86	(57%)
Other	5	(3%)
Clinical presentation (ACR components)		
Malar Rash	64	(42%)
Discoid Rash	22	(15%)
Oral Ulcers	57	(38%)
Photosensitivity	50	(33%)
Arthritis	106	(70%)
Serositis	66	(44%)
Renal Disorder	59	(39%)
Neurological Disorder	16	(11%)
Haematological Disorder	77	(51%)
Immunological Disorder	127	(84%)
Anti-nuclear Antibody positive	146	(97%)
Anti-nuclear antibodies ≥ 1280	112	(77%)
Anti-dsDNA positive	82	(54.3%)
Active disease at baseline (SLEDAI > 4)	62	(41%)
Adjusted mean SLEDAI (AMS) > 4	74	(51%)
Persistently active disease (PAD)	92	(62%)
**Serum cytokines at baseline**	**Median**	**[IQR] (range)**
IFN-CK score	0.27	[0.16, 0.57] (0.03, 3.0)
MIF (ng/mL)	6.7	[4.2, 12.1] (0.02, 111)

**Table 2:** Univariable associations of persistently active disease.

	Unadjusted OR	(95% CI)	p-value
Baseline IFN-CK score			
1^st^ quartile (lowest)	1.00		
2^nd^ quartile	1.28	(0.53, 3.06)	0.6
3^rd^ quartile	2.35	(0.90, 6.11)	0.08
4^th^ quartile	2.67	(1.04, 6.87)	0.04
Baseline MIF			
1^st^ quartile (lowest)	1.00		
2^nd^ quartile	1.44	(0.63, 3.33)	0.4
3^rd^ quartile	1.82	(0.71, 4.63)	0.2
4^th^ quartile	4.60	(1.49, 14.2)	<0.01
Ethnicity			
Non-Asians	1.00		
Asians	3.38	(1.61, 7.10)	<0.01

OR = Odds Ratio; CI = Confidence Interval.

**Table 3:** Characteristics of study population, stratified by ethnicity.

	Non-Asian (n = 91)		Asian (n = 60)		P value
	Median	[IQR] (range)	Median	[IQR] (range)	
Age at diagnosis (years)	46	[37,61] (21, 79)	37.5	[29.5, 45.5] (20, 75)	<0.01
Disease duration at recruitment (years)	8.6	[5.6, 17.6] (1.5, 36.6)	7.6	[3.6, 14.1] (1.6, 27.6)	0.07
Number of ACR criteria	5	[4,6] (4, 9)	5	[4, 6] (4, 9)	0.3
SLEDAI–2k (baseline)	4	[2,8] (0, 16)	4	[2, 7] (0, 20)	0.4
SLICC-SDI (baseline)	0	[0, 1] (0, 3)	0	[0, 1] (0, 7)	0.7
Number of visits	4	[2,7] (1, 26)	8	[4, 12] (1, 27)	<0.01
Length of follow up	2.8	[1.4, 3.5] (0, 4.6)	2.7	[1.5, 3.8] (0, 4.6)	0.8
	**n**	**%**	**n**	**%**	**P value**
Number of females	73	(80%)	54	(90%)	0.11
Clinical Presentation					
Malar Rash	34	(37%)	30	(50%)	0.12
Discoid Rash	15	(16%)	7	(12%)	0.4
Oral Ulcers	36	(40%)	21	(35%)	0.5
Photosensitivity	34	(37%)	16	(27%)	0.17
Arthritis	71	(78%)	35	(58%)	0.01
Serositis	42	(46%)	24	(40%)	0.4
Renal Disorder	27	(30%)	32	(53%)	<0.01
Neurological Disorder	9	(10%)	7	(12%)	0.7
Haematological Disorder	42	(46%)	35	(58%)	0.14
Immunological Disorder	72	(79%)	55	(92%)	0.04
Anti-nuclear Antibody positive	87	(96%)	59	(98%)	0.3
Prednisolone use	62	(69%)	55	(92%)	< 0.01
Adjusted mean SLEDAI (AMS) > 4	41	(47%)	33	(57%)	0.2
Persistent active disease (PAD)	46	(51%)	46	(78%)	< 0.01
Anti-nuclear antibodies ≥ 1280^1^	61	(71%)	51	(86%)	0.03
Anti-dsDNA Positive	43	(48%)	39	(65%)	0.05
ENA-positive	35	(41%)	44	(73%)	<0.01
**Serum Cytokines**	**Median**	**[IQR] (range)**	**Median**	**[IQR] (range)**	**P value**
MIF (ng/mL)	5.0	[3.4, 10.5] (0.02, 88)	8.7	[6.1, 16.3] (1.3, 111)	<0.01
IFN-CK score (0–3)	0.26	[0.16, 0.52] (0.03, 2.0)	0.32	[0.17, 0.63] (0.07, 3.0)	0.3

**Table 4:** Multivariable associations of persistently active disease with MIF and ethnicity.

	Adjusted OR*	(95% CI)	p-value
Baseline MIF			
1^st^ quartile (lowest)	1.00		
2^nd^ quartile	1.26	(0.53, 2.97)	0.6
3^rd^ quartile	1.39	(0.52, 3.70)	0.5
4^th^ quartile	3.62	(1.14, 11.5)	0.03
Ethnicity			
Non-Asians	1.00		
Asians	3.00	(1.39, 6.46)	<0.01

^*^Odds ratios were adjusted for MIF and ethnicity.

**Table 5:** Multivariable associations of persistently active disease with IFN-CK and ethnicity.

	Adjusted OR*	(95% CI)	p-value	
Baseline IFN-CK score				
1^st^ quartile (lowest)	1.00			
2^nd^ quartile	1.34	(0.54, 3.33)	0.5	
3^rd^ quartile	2.35	(0.87, 6.33)	0.09	
4^th^ quartile	2.53	(0.95, 6.75)	0.06	
Ethnicity				
Non-Asians	1.00			
Asians	3.30	(1.55, 7.01)	< 0.01	

^*^Odds ratio adjusted for IFN-CK and ethnicity.
