# Supplementary material for: Association of MIF, but not type I interferon-induced chemokines, with increased disease activity in Asian patients with systemic lupus erythematosus
Source: Sci Rep. 2016 Jul 25;6:29909. doi: 10.1038/srep29909 (PMC4958969; doi:10.1038/srep29909)

Association of MIF, but not type I interferon-induced chemokines, with increased disease activity in Asian patients with systemic lupus erythematosus

KL Connelly[1], R Kandane-Rathnayake[1], A Hoi[1], Mandana Nikpour[2], EF Morand[1*]

Supplementary Figure 1: Serum concentrations of MIF, and the interferon-induced chemokine score (IFNCK), were measured in 151 patients with SLE. Serum MIF and IFNCK were highly correlated (r=0.79, p<0.01).

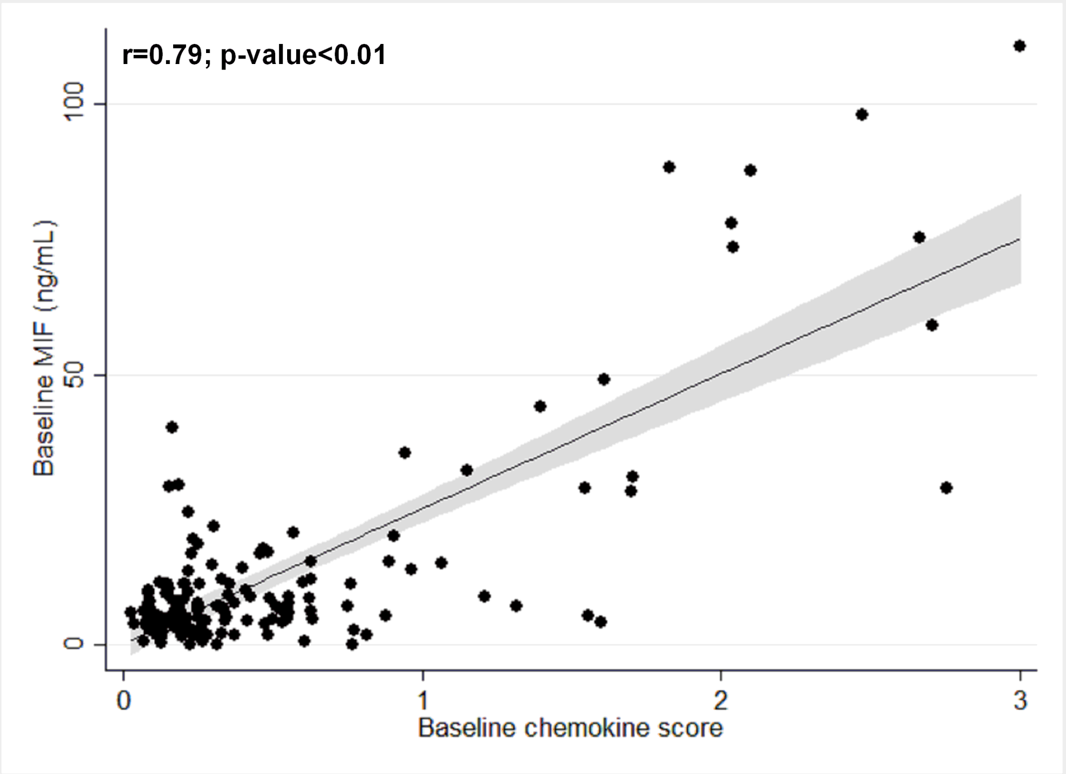

Supplement: Supplementary Figure S1 [file srep29909-s1.pdf]
