# Supplementary material for: Association of MIF, but not type I interferon-induced chemokines, with increased disease activity in Asian patients with systemic lupus erythematosus
Source: Sci Rep. 2016 Jul 25;6:29909. doi: 10.1038/srep29909 (PMC4958969; doi:10.1038/srep29909)
Supplement: Supplementary Table S1 [file srep29909-s2.pdf]

Association of MIF, but not type I interferon-induced chemokines, with increased disease activity in Asian patients with systemic lupus erythematosus

KL Connelly[1], R Kandane-Rathnayake[1], A Hoi[1], Mandana Nikpour[2], EF Morand[1*]

Supplementary Table 1: Association of organ activity with MIF IFN-CK

Baseline IFN-CK quartiles	Organ activity				Univariable associations		
	CNS -ve		CNS +ve				
	n	(%)	n	(%)	OR	(95% CI)	P-value
Quartile1 (lowest)	46	(33%)	3	(27%)	1.00		
Quartile 2	33	(24%)	3	(27%)	1.39	(0.26,7.34)	0.7
Quartile 3	28	(20%)	4	(36%)	2.19	(0.46,10.52)	0.3
Quartile 4	33	(24%)	1	(9%)	0.46	(0.05,4.67)	0.5
	vasculitis -ve		vasculitis +ve				
	n	(%)	n	(%)	OR	(95% CI)	P-value
Quartile1 (lowest)	48	(33%)	1	(17%)	1.00		
Quartile 2	34	(23%)	2	(33%)	2.82	(0.25,32.41)	0.4
Quartile 3	29	(20%)	3	(50%)	4.97	(0.49,50.01)	0.2
Quartile 4	34	(23%)	0				
	MSK -ve		MSK +ve				
	n	(%)	n	(%)	OR	(95% CI)	P-value
Quartile1 (lowest)	42	(40%)	7	(16%)	1.00		
Quartile 2	25	(24%)	11	(24%)	2.64	(0.91,7.69)	0.08
Quartile 3	19	(18%)	13	(29%)	4.11	(1.41,11.93)	<0.01
Quartile 4	20	(19%)	14	(31%)	4.20	(1.47,12.03)	<0.01
	renal -ve		renal +ve				
	n	(%)	n	(%)	OR	(95% CI)	P-value
Quartile1 (lowest)	36	(35%)	13	(27%)	1.00		
Quartile 2	27	(26%)	9	(18%)	0.92	(0.34,2.47)	0.9
Quartile 3	16	(16%)	16	(33%)	2.77	(1.08,7.08)	0.03
Quartile 4	23	(23%)	11	(22%)	1.32	(0.51,3.45)	0.6
	Cutaneous -ve		Cutaneous +ve				
	n	(%)	n	(%)	OR	(95% CI)	P-value
Quartile1 (lowest)	24	(34%)	25	(31%)	1.00		
Quartile 2	18	(25%)	18	(23%)	0.96	(0.41,2.27)	0.9
Quartile 3	15	(21%)	17	(21%)	1.09	(0.45,2.65)	0.9
Quartile 4	14	(20%)	20	(25%)	1.37	(0.57,3.32)	0.5
	Serositis -ve		Serositis +ve				
	n	(%)	n	(%)	OR	(95% CI)	P-value
Quartile1 (lowest)	47	(33%)	2	(22%)	1.00		

Quartile 2	34	(24%)	2	(22%)	1.38	(0.19,10.31)	0.8
Quartile 3	29	(20%)	3	(33%)	2.43	(0.38,15.43)	0.3
Quartile 4	32	(23%)	2	(22%)	1.47	(0.20,10.97)	0.7
	Serological -ve		Serological +ve				
	n	(%)	n	(%)	OR	(95% CI)	P-value
Quartile1 (lowest)	12	(46%)	37	(30%)	1.00		
Quartile 2	8	(31%)	28	(22%)	1.14	(0.41,3.15)	0.8
Quartile 3	3	(12%)	29	(23%)	3.14	(0.81,12.16)	0.1
Quartile 4	3	(12%)	31	(25%)	3.35	(0.87,12.96)	0.08
	Fever -ve		Fever +ve				
	n	(%)	n	(%)			
Quartile1 (lowest)	49	(33%)	0				
Quartile 2	33	(22%)	3	(100%)			
Quartile 3	32	(22%)	0				
Quartile 4	34	(23%)	0				
	Haem -ve		Haem +ve				
	n	(%)	n	(%)	OR	(95% CI)	P-value
Quartile1 (lowest)	43	(34%)	6	(26%)	1.00		
Quartile 2	34	(27%)	2	(9%)	0.42	(0.08,2.22)	0.3
Quartile 3	24	(19%)	8	(35%)	2.39	(0.74,7.70)	0.14
Quartile 4	27	(21%)	7	(30%)	1.86	(0.56,6.12)	0.3
