# Supplementary material for: Association of MIF, but not type I interferon-induced chemokines, with increased disease activity in Asian patients with systemic lupus erythematosus
Source: Sci Rep. 2016 Jul 25;6:29909. doi: 10.1038/srep29909 (PMC4958969; doi:10.1038/srep29909)
Supplement: Supplementary Table S2 [file srep29909-s3.pdf]

**Association of MIF, but not type I interferon-induced chemokines, with increased disease activity in Asian patients with systemic lupus erythematosus**

KL Connelly[1], R Kandane-Rathnayake[1], A Hoi[1], Mandana Nikpour[2], EF Morand[1*]

**Supplementary Table 2: Association of organ activity with MIF**

| Baseline MIF quartiles | Organ Activity | | | | Univariate associations | | |
|---|---|---|---|---|---|---|---|
| | CNS -ve | | CNS +ve | | | | |
| | n | (%) | n | (%) | OR | (95% CI) | P-value |
| Quartile1 (lowest) | 43 | (31%) | 4 | (36%) | 1.00 | | |
| Quartile 2 | 42 | (30%) | 2 | (18%) | 0.51 | (0.09,2.95) | 0.4 |
| Quartile 3 | 29 | (21%) | 3 | (27%) | 1.11 | (0.23,5.34) | 0.9 |
| Quartile 4 | 26 | (19%) | 2 | (18%) | 0.83 | (0.14,4.83) | 0.8 |
| | Vasculitis -ve | | Vasculitis +ve | | | | |
| | n | (%) | n | (%) | OR | (95% CI) | P-value |
| Quartile1 (lowest) | 45 | (31%) | 2 | (33%) | 1.00 | | |
| Quartile 2 | 44 | (30%) | 0 | | | | |
| Quartile 3 | 28 | (19%) | 4 | (67%) | 3.21 | (0.55,18.71) | 0.2 |
| Quartile 4 | 28 | (19%) | 0 | | | | |
| | MSK -ve | | MSK +ve | | | | |
| | n | (%) | n | (%) | OR | (95% CI) | P-value |
| Quartile1 (lowest) | 32 | (30%) | 15 | (33%) | 1.00 | | |
| Quartile 2 | 34 | (32%) | 10 | (22%) | 0.63 | (0.25,1.60) | 0.3 |
| Quartile 3 | 22 | (21%) | 10 | (22%) | 0.97 | (0.37,2.55) | 0.9 |
| Quartile 4 | 18 | (17%) | 10 | (22%) | 1.19 | (0.44,3.18) | 0.7 |
| | Renal -ve | | Renal +ve | | | | |
| | n | (%) | n | (%) | OR | (95% CI) | P-value |
| Quartile1 (lowest) | 35 | (34%) | 12 | (24%) | 1.00 | | |
| Quartile 2 | 30 | (29%) | 14 | (29%) | 1.36 | (0.55,3.39) | 0.5 |
| Quartile 3 | 18 | (18%) | 14 | (29%) | 2.27 | (0.87,5.91) | 0.09 |
| Quartile 4 | 19 | (19%) | 9 | (18%) | 1.38 | (0.49,3.87) | 0.5 |
| | Cutaneous -ve | | Cutaneous +ve | | | | |
| | n | (%) | n | (%) | OR | (95% CI) | P-value |
| Quartile1 (lowest) | 28 | (39%) | 19 | (24%) | 1.00 | | |
| Quartile 2 | 23 | (32%) | 21 | (26%) | 1.35 | (0.59,3.09) | 0.5 |
| Quartile 3 | 11 | (15%) | 21 | (26%) | 2.81 | (1.11,7.16) | 0.03 |
| Quartile 4 | 9 | (13%) | 19 | (24%) | 3.11 | (1.16,8.32) | 0.02 |
| | Serositis -ve | | Serositis +ve | | | | |
| | n | (%) | n | (%) | OR | (95% CI) | P-value |
| Quartile1 (lowest) | 45 | (32%) | 2 | (22%) | 1.00 | | |
| Quartile 2 | 43 | (30%) | 1 | (11%) | 0.52 | (0.05,5.98) | 0.6 |

| | | | | | | | |
|---|---|---|---|---|---|---|---|
| Quartile 3 | 28 | (20%) | 4 | (44%) | 3.21 | (0.55,18.71) | 0.19 |
| Quartile 4 | 26 | (18%) | 2 | (22%) | 1.73 | (0.23,13.03) | 0.6 |
| | Serological -ve | | Serological +ve | | | | |
| | n | (%) | n | (%) | OR | (95% CI) | P-value |
| Quartile1 (lowest) | 10 | (38%) | 37 | (30%) | 1.00 | | |
| Quartile 2 | 8 | (31%) | 36 | (29%) | 1.22 | (0.43,3.43) | 0.7 |
| Quartile 3 | 7 | (27%) | 25 | (20%) | 0.97 | (0.32,2.87) | 0.9 |
| Quartile 4 | 1 | (4%) | 27 | (22%) | 7.30 | (0.88,60.47) | 0.06 |
| | Fever -ve | | Fever +ve | | | | |
| | n | (%) | n | (%) | OR | (95% CI) | P-value |
| Quartile1 (lowest) | 46 | (31%) | 1 | (33%) | 1.00 | | |
| Quartile 2 | 44 | (30%) | 0 | | | | |
| Quartile 3 | 31 | (21%) | 1 | (33%) | 1.48 | (0.09,24.62) | 0.8 |
| Quartile 4 | 27 | (18%) | 1 | (33%) | 1.70 | (0.10,28.36) | 0.7 |
| | Heam -ve | | Heam +ve | | | | |
| | n | (%) | n | (%) | OR | (95% CI) | P-value |
| Quartile1 (lowest) | 43 | (34%) | 4 | (17%) | 1.00 | | |
| Quartile 2 | 38 | (30%) | 6 | (26%) | 1.70 | (0.45,6.47) | 0.4 |
| Quartile 3 | 25 | (20%) | 7 | (30%) | 3.01 | (0.80,11.31) | 0.1 |
| Quartile 4 | 22 | (17%) | 6 | (26%) | 2.93 | (0.75,11.49) | 0.1 |
